# Complete Genome Analysis of African Swine Fever Virus Isolated from Wild Boar, India, 2021

**DOI:** 10.3201/eid3108.250083

**Published:** 2025-08

**Authors:** Dhanapal Senthilkumar, Katherukamem Rajukumar, Govindarajulu Venkatesh, Fateh Singh, Gopal Sarkar, Jaswant Patel, Suman Mishra, Rohit Sahu, Nourin Khan, C. Neihthangpuii, Esther Lalzolian, Vijendra Pal Singh, Aniket Sanyal

**Affiliations:** ICAR, National Institute of High Security Animal Diseases, Bhopal, India (D. Senthilkumar, K. Rajukumar, G. Venkatesh, F. Singh, G. Sarkar, J. Patel, S. Mishra, R. Sahu, N. Khan, V.P. Singh, A. Sanyal); Government of Mizoram, Aizawl, India (C. Neihthangpuii, E. Lalzoliani)

**Keywords:** African swine fever virus, viruses, wild boar, complete genome, India

## Abstract

Complete genome analysis of African swine fever virus isolated from a wild boar in Mizoram, India, revealed ≈99% nucleotide identity with those of domestic pig origin but with unique mutations. A One Health approach toward food security necessitates awareness among veterinary and public health professionals on virus evolution and domestic–wild pig transmission.

African swine fever (ASF) is a devastating disease affecting pigs, with death rates reaching 100%. Wild boars (*Sus scrofa*), warthogs (*Phacochoerus aethiopicus*), and bushpigs (*Potamochoerus porcus*) can act as asymptomatic carriers, contributing to virus persistence in a sylvatic cycle (*1*). Soft ticks of the genus *Ornithodoros* further complicate ASF epidemiology. The disease is caused by ASF virus (ASFV), a large, double-stranded DNA virus belonging to the genus *Asfivirus* in the Asfarviridae family. The ASFV genome range is 171–193 kb, featuring inverted terminal repeats (ITRs) at both ends. Different ASFV genotypes are based on the 3′ end of the *B646L* gene; genotype II predominates in Asia, Europe, Oceania, and the Americas. Recent emergence of novel recombinant genotype I/II strains in China and Vietnam (*2*,*3*) is of great concern. 

ASF was first reported in India in 2020 after outbreaks in domestic pigs in northeastern states (*4*). Outbreaks in wild boars have been documented in Assam, Karnataka, and Tamilnadu states (*5*,*6*). ASFVs circulating in India belong to genotype II and intergenic region (IGR) subcluster II. Complete genome sequencing of Indian ASFV isolates of domestic pig origin revealed unique mutations in the *MGF 360–11L, MGF 505–4R, K205R*, and *B263R* genes (*7*). We analyzed the complete genome of ASFVs isolated after ASF outbreaks affecting domestic pigs and wild boars in Mizoram, India, in August 2021.

We collected 40 samples from dead domestic pigs and those suspected of having ASF (Appendix Figure 1); 38 of those samples and a dead wild boar tested positive for ASF genome by quantitative PCR and were confirmed by virus isolation. We grew, processed for viral enrichment, and sequenced 1 ASFV isolate (MZ/21/PO-324) of wild boar origin and 1 isolate (MZ/21/PO-314) of domestic pig origin. We submitted nucleotide sequences to GenBank (accession nos. PV023909 and PV023910) (Appendix).

The wild boar ASFV genome comprised 190,489 bp with ITRs at the 5′ end (1,597 bp) and 3′ end (1,122 bp), whereas the ASFV genome from domestic pig measured 189,390 bp and had a 5′ end ITR of 422 bp and 3′ end ITR of 1,150 bp. Comparative analysis showed 99.93% nucleotide identity between those isolates (Appendix Figure 2). Phylogenetic analysis placed the ASFV in India within genotype II clade 2.2.2, alongside genotype II viruses reported during 2007–2023 across diverse regions. Within p72 genotype II viruses, isolates from India formed a distinct cluster with isolate ASFV/Wuhan/2019 (Figure 1). *EP402R* gene–based serogrouping confirmed isolates from India as part of serogroup 8, consistent with hemadsorption-positive viruses (Appendix Figure 3). There was an insertion of an additional tandem repeat sequence in the extragenic region between *I73R* and *I329L* (Appendix Figure 4), aligning with intergenic region II cluster isolates of genotype II. Central variable region analysis of the *B602L* gene indicated similarity to the Georgia 2007/1 central variable region I variant (*8*).

**Figure 1:**
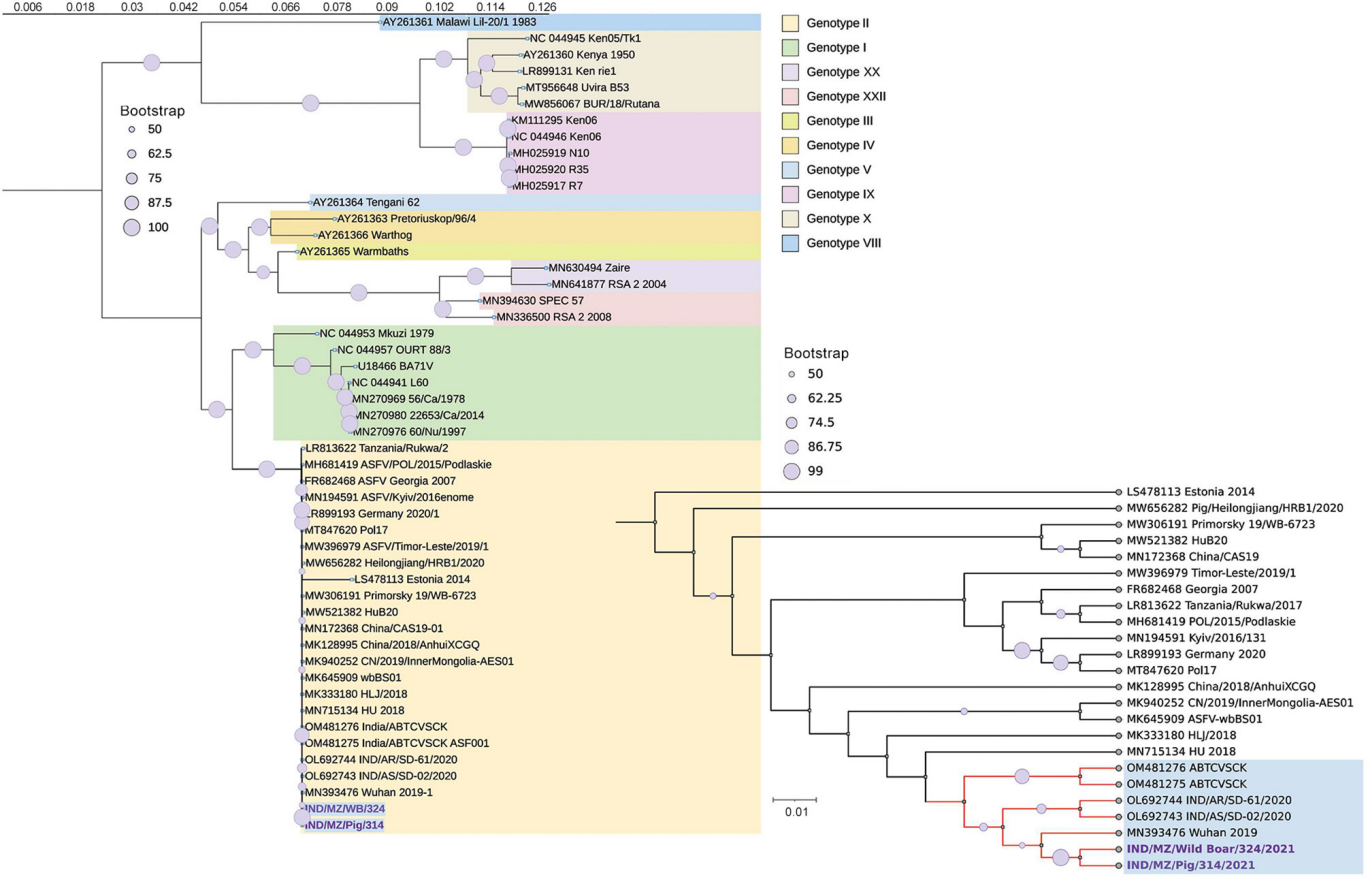
Phylogenetic tree generated from complete genome analysis of ASFV isolated from wild boar and domestic pig, India, 2021. The maximum-likelihood tree was MAFFT aligned (https://mafft.cbrc.jp/alignment/software) by using a general time-reversible plus gamma model in RAxMLGUI version 2.0 (https://sourceforge.net/projects/raxmlgui) and shows the relationship between ASFVs from India and other ASFVs including p72 genotype II. Enlarged area at bottom right shows detail of isolates from this study (bold text) and close reference sequences. GenBank accession numbers are provided. Scale bar indicates the number of expected substitutions per site (tree rooted at mid-point). ASFV, African swine fever virus.

We observed multiple nucleotide insertions and deletions (Appendix Table 1) in the genome sequence of the wild boar isolate compared with isolate ASFV-Georgia/2007, leading to frame shift mutations in *DP60R* and *ASFV-GACD 190* genes, amino acid additions in *ASFV GACD-00300* and *ASFV GACD-00350* genes, and protein truncations in immune-modulatory genes, *MGF 110–7L, MGF 110–10L, MGF 110–14L, MGF 110–13Lb, I196L, B475L,* and *MGF 360–21R*, of which the last 3 mutations were unique to the wild boar isolate (Appendix). A 50-nt deletion in the *MGF 360–21R* gene resulted in a truncated protein of 327 aa. We did not observe that deletion in the ASFV isolate of domestic pig origin, and the deletion was unique to the wild boar isolate reported in this study. Further analyses and multiple sequence alignment of *MGF-360–21R* gene of ASFV isolates obtained from wild boar, warthog, and domestic pigs across different countries revealed that the gene is particularly susceptible to mutations during replication in wild boars compared with domestic pigs (Figure 2) and causes truncations at the carboxyl terminus of the encoded protein. Those observations reflect the role of the *MGF-360–21R* gene in evolutionary adaptations of ASFV in wild boar populations.

**Figure 2:**
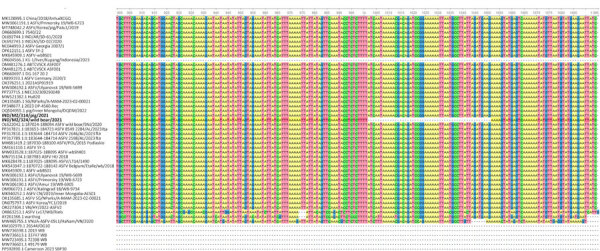
Complete genome analysis of ASFV isolated from wild boar and domestic pig, India, 2021. A 50-nt deletion is observed in the *MGF-360–21R* gene of the ASFV isolate obtained from a wild boar in Mizoram, India, compared with ASFV isolates derived from domestic pigs in India. Bold isolates are from this study. GenBank accession numbers are provided. ASFV, African swine fever virus.

A comparative analysis of genotype II ASFV revealed 20 single-nucleotide polymorphisms comprising 16 nonsynonymous and 4 synonymous mutations across 18 open reading frames (Appendix Figure 5). Key nonsynonymous mutations included K32E in *ASFV GACD 300*, P406L in *EP1242L,* R188K in K205R, and Q104H in *E199L*. The proteins encoded by *K205R* and *E199L* genes are noted to interact with host proteins, potentially activating cellular responses such as unfolded protein response and autophagy (*9*,*10*).

In conclusion, ASFV sequences from both hosts showed ≈99% identity and highlighted transmission between domestic and wild pigs. However, we identified unique genetic variations in ASFVs isolated from wild boar, which may influence viral interactions with host cellular machinery. Our findings highlight the critical role of wild boars in ASF epidemiology and underscore the need for veterinary, wildlife and public health authorities to be aware of transmission dynamics between domestic and wild pigs and viral evolution, with implications for viral survival, immune modulation, and control strategies. 

Appendix Additional information for complete genome analysis of African swine fever virus isolated from wild boar, India, 2021
