# Supplementary material for: Complete Genome Analysis of African Swine Fever Virus Isolated from Wild Boar, India, 2021
Source: Emerg Infect Dis. 2025 Aug;31(8):1665–8. doi: 10.3201/eid3108.250083 (PMC12309776; doi:10.3201/eid3108.250083)
Supplement: Appendix — Additional information for complete genome analysis of African swine fever virus isolated from wild boar, India, 2021 [file 25-0083-Techapp-s1.pdf]

Article DOI: https://doi.org/10.3201/eid3108.250083

EID cannot ensure accessibility for supplementary materials supplied by authors. Readers who have difficulty accessing supplementary content should contact the authors for assistance.

Complete Genome Analysis of African Swine Fever Virus Isolated from Wild Boar, India, 2021

Appendix

Section 1

Samples and Testing

During August 2021, we received whole blood, serum, and tissue samples collected from suspected and dead pigs from different parts of Mizoram and 1 wild boar sample collected from a dead carcass found in forest area of Serchhip district of Mizoram. Tissue, serum, and plasma separated from the whole blood samples were tested by real-time qPCR and subjected to virus isolation. The number of samples tested from various districts of Mizoram state during this outbreak is presented in Appendix Figure 1. Viral nucleic acid was extracted from the samples using QIAamp Viral RNA Mini Kit (Qiagen, Germany) following the manufacturer's recommendation. Viral nucleic acid was eluted in 50 µL of elution buffer of the kit. Nucleic acid extracted from the suspected samples was tested by real-time PCR for the detection of ASFV genome as described previously (*1*) with suitable modifications using Premix Ex Taq (Probe qPCR, Takara Bio Inc., Japan). Briefly, each 20-µL reaction mix consisted of 1× reaction buffer, 0.4 µM of each forward (5′-CCCAGGRGATAAAATGACTG-3′) and reverse (5′-CACTRGTTCCCTCCACCGATA-3′) primer, 0.1 µM of probe (5′-FAM-TCCTGGCCRACCAAGTGCTT-BHQ1–3′), 5 µL of template DNA, and nuclease free water to make up the volume. The reactions were carried out in CFX96 Real-Time PCR System (Bio-Rad, USA) with the following cycling conditions: 1 cycle at 95°C for 2 minutes, followed by 45 cycles at 95°C for 10 seconds and 60°C for 30 seconds. Samples showing proper amplification curves with a Ct value <40 were considered positive.

Virus Isolation

The processed samples (10% tissue homogenates and serum) were inoculated onto porcine pulmonary alveolar macrophage (PAM) cultures following a previously described protocol (*2*) with appropriate modifications. Briefly, cryopreserved PAM harvested from the lungs of 5-week-old piglets were thawed, washed, and resuspended in RPMI-1640 medium (Sigma, USA). The cells were seeded in 96-well plates at a concentration of 2×10^5 cells per well in 100 µL volume. Samples were inoculated in triplicate at 1/10, 1/50, and 1/100 dilutions. Three wells served as uninoculated controls. On the following day, 20 µL of freshly prepared 1% swine red blood cell suspension in RPMI-1640 medium/PBS was added to each well. The plates were incubated at 37°C in a CO_2 incubator and monitored for 5–7 days for the development of hemadsorption (HAD) or cytopathic effects indicative of ASFV presence.

Viral Genome Enrichment

Two ASF viruses [one each isolated from domestic pig (IND/MZ/314/2021) and wild boar (IND/MZ/324/2021)] were selected for complete genome sequencing. The virus isolates were bulk propagated and processed for viral genome enrichment according to the protocol described by Alexander et al. (*3*), with suitable modifications. Briefly, infected cells were subjected to 3 freeze-thaw cycles to release viral particles. The cell lysates were clarified by centrifugation at 3,250 × *g* for 20 minutes at 4°C. The supernatant was collected and mixed with 100 mM $MgSO_4$ in 50 mM Tris-HCl buffer (pH 7.5). A 50% (w/v) solution of polyethylene glycol (PEG) 6000 was prepared in NT buffer (150 mM NaCl and 50 mM Tris-HCl) and added to the supernatant to achieve a final concentration of 10% PEG. The mixture was incubated overnight with constant stirring at 4°C. The solution was centrifuged at 3,250 × *g* for 20 minutes at 4°C. The supernatant was discarded, and the precipitated pellet was resuspended in 500 µL of cold NT buffer. The viral pellet was treated with DNase (70 units) at 37°C for 1 hour to remove host genomic DNA. DNase activity was stopped by incubation with 50 mM EDTA (final concentration) at 65°C for 10 minutes. The pellet was subsequently treated with RNase (40 µg/mL), proteinase K (200 µg/mL), and 1% sodium dodecyl sulfate (SDS) at 37°C for 18 hours. DNA was extracted from the treated pellet using the phenol-chloroform extraction method (*4*). The DNA was then quantified and stored at −80°C until further use.

Whole-Genome Sequencing

The quality and quantity of the genomic DNA extracted from the enriched virus pellet were assessed using a nano-spectrophotometer (Eppendorf, Germany). Real-time PCR was carried out to confirm the presence of ASFV genome. Complete genome sequencing was performed by preparing a sequencing library for each sample using the Nextera XT DNA library preparation kit (Illumina, San Diego, CA, USA), followed by 2 × 150 NextSeq500 paired-end sequencing on the NextSeq500 sequencing system (Illumina, USA).

The next-generation sequencing of ASFV isolates from wild boar and domestic pig yielded 20,823,144 and 6,180,140 raw reads, respectively. Quality control of the raw reads was performed using FastQC v0.11.9 (*5*), which provided a comprehensive overview of read quality, including total bases, read count, GC content, and other basic statistics. Low-quality reads, with Phred scores below 30, as well as adaptor sequences, were removed using Trimmomatic v0.38 (*6*) to ensure high-quality data for downstream analysis. De novo assembly of the quality-filtered reads was carried out using SPAdes v3.15.2 (*7*), generating scaffold contigs. Contigs smaller than 200 kb were subjected to Blastn analysis to identify the closest matching ASFV sequence. The trimmed reads were then aligned to the best-matched ASFV genome (GenBank accession no. OL697244) using HISAT-2 /BWA-MEM software (*8*) to assemble the complete genome. Gaps or regions with read coverage depth below 50 were resolved through PCR amplification, using custom primers (Appendix Table 2), followed by Sanger sequencing. Gene annotation of the assembled genomes was performed using the Genome Annotation Transfer Utility (GATU) software (*9*). The final complete genome sequences were compiled for further analysis.

Phylogenetic and Single-Nucleotide Variant Analyses

Complete genome sequences of the ASF virus isolates from a domestic pig (IND/MZ/314/2021) and a wild boar (IND/MZ/324/2021) were aligned with 48 additional complete genome sequences retrieved from the NCBI GenBank database. For serogroup classification, the EP402R gene from these isolates was aligned with other sequences obtained from the GenBank database. All sequence alignments were performed using the MAFFT software (v7.487) (*10*), employing default parameters. Phylogenetic analysis was conducted using the Randomized Axelerated Maximum Likelihood (RAxML GUI v2.0.13) software (*11*). Maximum likelihood (ML) phylogenetic trees were constructed from the aligned sequences under the general time reversible (GTR) model with gamma distribution to account for rate of variation across sites. To assess the robustness of the tree, bootstrap analysis was performed with 1,000 pseudo-replicates. The phylogenetic trees were visualized and annotated using iTOL v6 (Interactive Tree of Life) software (*12*).

Single-nucleotide polymorphism (SNP) analysis was carried out by comparing with other complete genome sequences reported in India and other countries: IND/AS/SD-02/2020 (OL692743), IND/AR/SD-61/2020 (OL692744), IND/ABTCVSCK (OM481275), IND/ABTCVSCK (OM481276), China/2018/Anhui (MK128995.1), ASFV Wuhan 2019–1 (MN393476.1), and ASFV Georgia 2007/1 (NC_044959.2) using mpileup utility of Samtools (v0.1.18) from sorted BAM files (*13*). The percentage of nucleotide identity were generated from aligned sequences using Qiagen CLC genomics workbench version 5.5.4.

Section 2

Nucleotide Insertions and Deletions

Alignment of the complete genome sequence of ASFV isolated from wild boar with the reference strain Georgia/2007 revealed notable nucleotide variations. Seven single-nucleotide insertions [1 G (at position 103315), 1 T (position 468 in *DP60R* gene), 2 Cs (position 1381 in IR, 10595 in *MGF 110–7L* gene) 3 As (at 4197, 168750 and 190372)], a double nucleotide insertion (TA) at position 74327, 2 triple nucleotide insertions (GGG) at 17837 and 21796, a 6-nucleotide insertion (TAAAAT) at 17936 (*ASFV G ACD 00300*), and a 10-nucleotide insertion (TATATAGGAA) at 173381 were identified in the wild boar ASFV genome.

Five single-nucleotide deletions (2A, 3T) were observed at positions 2958, 6774, 12568 (*ASFV G ACD 00190* gene), 21786, and 176021 (*I196L* gene). Other deletions included a 2-nucleotide deletion at 19791, a 12-nucleotide deletion at 11933, 4-nucleotide deletions at 14224 and 15665 (*MGF 110–13Lb*), a 3-nucleotide deletion at 19998 (*ASFV G ACD 00350*), a 9-nucleotide deletion at 100436 (*B475L* gene) and a 50-nt deletion at 188949 (*MGF 360–21R* gene).

These insertions and deletions resulted in frame shift mutations affecting amino acid expression in *DP60R* and *ASFV-GACD 190* genes. Truncations could be predicted in proteins encoded by *MGF 110–7L* gene (due to insertion of 1 extra G at position 133), *MGF 110–10L* and *MGF 110–14L* fusion gene (due to a 4-nucleotide deletion at position 343), and *MGF 110–13Lb*

gene (due to a 4-nucleotide deletion at position 363). Protein truncations observed in these genes may affect immune-modulatory functions (*14*).

Other predicted changes included the addition of 2 amino acids in the carboxyl end of the *ASFV GACD-00300* protein and 1 additional amino acid in *ASFV GACD-00350*. The 9-nucleotide deletion in the B475L gene led to the loss of 3 amino acids in its amino-terminal region, while a single nucleotide deletion at position 586 of *I196L* gene caused protein truncation, potentially impacting its immune evasion function (*15*). The 50-nt deletion (from positions 972 to 1021) observed in the *MGF 360–21R* gene resulted in a truncated protein of 327 aa instead of 357 aa.

Single-Nucleotide Variant Analysis

A comparative analysis between ASFV sequences from wild boar and the reference Georgia/2007 strain identified 15-nt differences, including 5 within the 5′ ITR, 2 in intergenic regions, and 8 within genic regions (Appendix Table 1). Further analysis of important genotype II ASF viruses revealed 20 SNP, comprising 16 nonsynonymous and 4 synonymous SNP distributed across 18 ORFs (Appendix Figure 5). Synonymous SNPs were observed in 4 ORFs: 5′ ITR *ASFV G ACD 01990*, *M1249L*, *B438L*, and 3′ ITR *ASFV G ACD 01990*.

Nonsynonymous SNPs were present in 14 ORFs, including *ASFV G ACD 300*, *MGF 360–11L*, *MGF 505–4R*, *MGF 505–9R*, *K205R*, *EP1242L*, *C717R*, *B263R*, *G1211R*, *O174L*, *NP419L*, *E199L*, *I267L*, and *MGF 360–21R*. Notably, unique SNPs in the wild boar isolate were identified in specific ORFs relative to previously reported Indian isolates. Key mutations included a synonymous SNP in 5′ ITR *ASFV G ACD 1990*, a nonsynonymous mutation K32E in *ASFV G ACD 300*, and 2 additional nonsynonymous changes R188K and Q104H in *K205R* and *E199L* genes, respectively. A synonymous mutation was also detected in the 3′ ITR *ASFV G ACD 01990*.

Phylogenetic Analysis

A comprehensive phylogenetic analysis of complete ASFV genomes, including Indian isolates and 44 additional ASFV sequences retrieved from GenBank, placed the Indian isolates within clade 2.2.2, alongside genotype II viruses reported from 2007 to 2023 across diverse regions such as Georgia, Tanzania, China, Vietnam, Poland, Ukraine, East Timor Leste, Estonia, and Germany. This clustering highlights the relatedness of Indian ASFV isolates to other globally circulating genotype II strains.

Further, a focused phylogenetic analysis of p72 genotype II viruses only revealed clustering of Indian isolates from Arunachal Pradesh, Assam, Meghalaya, and Mizoram in a discrete clade along with ASFV/Wuhan/2019. These findings underscore the genetic distinctiveness of the Indian ASF strains among the genotype II viruses, within the broader landscape of ASFV diversity and evolution.

A phylogenetic analysis based on partial *B602L* gene sequences of ASFV isolated from Mizoram (*16*) and Kerala states (*17*) reported to formed separate clusters, distinct from other Indian isolates reported from Assam and Arunachal Pradesh. This was linked to 1 amino acid mutation in the carboxy terminus region of *B602L* gene However, our analysis did not show any

nucleotide variation in the analyzed region of *B602L* among Indian isolates. The reported nucleotide variations were located within the primer binding region (*18*), emphasizing the need for complete genome sequencing of those earlier isolates to accurately assess the genetic divergence among Indian ASFV strains.

Serogrouping Based on the EP402R Gene Analysis

Phylogenetic analysis of the *EP402R* gene of Indian ASFV isolates, alongside sequences from various serogroups, revealed a distinct clustering of all Indian isolates with other Genotype II viruses within serogroup 8 (Appendix Figure 3) (*19*). This clustering pattern aligns with the observed hemadsorption phenotype in HAD assay, suggesting that the CD2v protein encoded by EP402R remains functionally intact, with no observed mutations in this gene among Indian isolates. This implies that the EP402R gene in Indian isolates is conserved, likely maintaining its role in immune interactions consistent with other serogroup 8 viruses.

References

1. Fernández-Pinero J, Gallardo C, Elizalde M, Robles A, Gómez C, Bishop R, et al. Molecular diagnosis of African swine fever by a new real-time PCR using universal probe library. Transbound Emerg Dis. 2013;60:48–58. PubMed https://doi.org/10.1111/j.1865-1682.2012.01317.x

2. Borca MV, Ramirez-Medina E, Silva E, Vuono E, Rai A, Pruitt S, et al. Development of a highly effective African swine fever virus vaccine by deletion of the I177L gene results in sterile immunity against the current epidemic Eurasia strain. J Virol. 2020;94:2017–9. PubMed https://doi.org/10.1128/JVI.02017-19

3. Alexander MR, Rootes CL, van Vuren PJ, Stewart CR. Concentration of infectious SARS-CoV-2 by polyethylene glycol precipitation. J Virol Methods. 2020;286:113977. PubMed https://doi.org/10.1016/j.jviromet.2020.113977

4. Sambrook J, Russell DW. Purification of nucleic acids by extraction with phenol: chloroform. CSH Protoc. 2006;2006:pdb.prot4455. PubMed https://doi.org/10.1101/pdb.prot4455

5. Andrews S, Krueger F, Segonds-Pichon A, Biggins L, Krueger C, Wingett S. FastQC: a quality control tool for high throughput sequence data [cited 2023 Jun 10]. https://www.bioinformatics.babraham.ac.uk/projects/fastqc.

6. Bolger AM, Lohse M, Usadel B. Trimmomatic: a flexible trimmer for Illumina sequence data. Bioinformatics. 2014;30:2114–20. PubMed https://doi.org/10.1093/bioinformatics/btu170

7. Bankevich A, Nurk S, Antipov D, Gurevich AA, Dvorkin M, Kulikov AS, et al. SPAdes: a new genome assembly algorithm and its applications to single-cell sequencing. J Comput Biol. 2012;19:455–77. PubMed https://doi.org/10.1089/cmb.2012.0021

8. Kim D, Langmead B, Salzberg SL. HISAT: a fast spliced aligner with low memory requirements. Nat Methods. 2015;12:357–60. PubMed https://doi.org/10.1038/nmeth.3317

9. Tcherepanov V, Ehlers A, Upton C. Genome annotation transfer utility (GATU): rapid annotation of viral genomes using a closely related reference genome. BMC Genomics. 2006;7:150. PubMed https://doi.org/10.1186/1471-2164-7-150

10. Katoh K, Rozewicki J, Yamada KD. MAFFT online service: multiple sequence alignment, interactive sequence choice and visualization. Brief Bioinform. 2019;20:1160–6. PubMed https://doi.org/10.1093/bib/bbx108

11. Kozlov AM, Darriba D, Flouri T, Morel B, Stamatakis A. RAxML-NG: a fast, scalable and user-friendly tool for maximum likelihood phylogenetic inference. Bioinformatics. 2019;35:4453–4455. PubMed https://doi.org/10.1093/bioinformatics/btz305

12. Letunic I, Bork P. Interactive Tree of Life (iTOL) v5: an online tool for phylogenetic tree display and annotation. Nucleic Acids Res. 2021;49:W293–6. PubMed https://doi.org/10.1093/nar/gkab301

13. Li H, Handsaker B, Wysoker A, Fennell T, Ruan J, Homer N, et al. The sequence alignment/map format and SAMtools. Bioinformatics. 2009;25:2078–2079. PubMed https://doi.org/10.1093/bioinformatics/btp352

14. Zhong H, Fan S, Du Y, Zhang Y, Zhang A, Jiang D, et al. African swine fever virus MGF110–7L induces host cell translation suppression and stress granule formation by activating the PERK/PKR-eIF2α pathway. Microbiol Spectr. 2022;10:e0328222. PubMed https://doi.org/10.1128/spectrum.03282-22

15. Huang Z, Mai Z, Kong C, You J, Lin S, Gao C, et al. African swine fever virus pB475L evades host antiviral innate immunity via targeting STAT2 to inhibit IFN-I signaling. J Biol Chem. 2024;300:107472. PubMed https://doi.org/10.1016/j.jbc.2024.107472

16. Rajkhowa TK, Kiran J, Hauhnar L, Zodinpui D, Paul A, Sagolsem S. Molecular detection and characterization of African swine fever virus from field outbreaks in domestic pigs, Mizoram, India. Transbound Emerg Dis. 2022;69:e1028–36. PubMed https://doi.org/10.1111/tbed.14384

17. Hiremath J, Hemadri D, Nayakvadi S, Kumar C, Gowda CSS, Sharma D, et al. Epidemiological investigation of ASF outbreaks in Kerala (India): detection, source tracing and economic implications. Vet Res Commun. 2024;48:827–37. PubMed https://doi.org/10.1007/s11259-023-10254-3

18. Nix RJ, Gallardo C, Hutchings G, Blanco E, Dixon LK. Molecular epidemiology of African swine fever virus studied by analysis of four variable genome regions. Arch Virol. 2006;151:2475–94. PubMed https://doi.org/10.1007/s00705-006-0794-z

19. Qu H, Ge S, Zhang Y, Wu X, Wang Z. A systematic review of genotypes and serogroups of African swine fever virus. Virus Genes. 2022;58:77–87. PubMed https://doi.org/10.1007/s11262-021-01879-0

Appendix Table 1. Comparison of nucleotide variations observed in Indian and Chinese ASFV isolates with reference to Georgia/2007*

Position per GA/2007	Region	GA/2007 (FR682468.2)	Ind/MZ/WB/324	Ind/MZ/314	Ind/AR/SD-61/2020 (OL692744)	Ind/AS/SD2/2020 (OL692743)	Ind/ABTCVSCK ASF001 (OM481275)	Ind/ABTCVSCK (OM481276)	China/2018/AnhuiXCGQ (MK128995)	Wuhan 2019–1 (MN393476)
27	5′ ITR	T	A	A	A	A	A	A	A	A
33	5′ ITR	C	G	G	G	G	G	G	G	G
42	5′ ITR	T	C	C	C	C	C	C	C	C
121	5′ ITR	G	C	C	C	C	C	C	C	C
129	5′ ITR	A	T	T	T	T	T	T	T	T
468	A (DP60R)*	A	AT	AT	AT	AT	AT	AT	A	AT
742	ASFV G ACD 01990	G	T	T	T	T	T	T	T	T
1381	IR	ACC	ACCC	ACCC	ACCC	ACCC	ACCC	ACCC	ACCC	ACCC
2958	IR	TT	T	T	T	T	T	T	T	T
4197	IR	C	CA	CA	CA	CA	CA	CA	CA	CA
6774	IR	CT	C	C	C	C	C	C	C	C
7781	IR	G	G	G	A	A	A	A	A	A
10595	MGF 110–7L	A	AC	AC	A	A	A	A	A	A
11933	IR	TTCTATTAAAAAA	T (Deletion of 14 nt)	TTCTATTAAAAAA	TTCTATTAAAAAA	TTCTATTAAAAAA	TTCTATTAAAAAA	TTCTATTAAAAAA	TTCTATTAAAAAA	TTCTATTAAAAAA
12568	ASFV G ACD 00190	GA	G	G	G	G	GA	GA	GA	GA
14224	MGF 110–11-L-MGF110–14L fusion	ACCCC	A	A	ACC	ACCC	ACCCC	ACCCC		
15665	MGF 110–13Lb	ACCCC	A	ACCC	A	A	ACCCCCCC	ACCCCCCC		
17837	Intergenic region	C	CGGG	CG	CG	CG	CG	CG	C	C
17936	ASFV G ACD 00300	A	ATAAAAT	A	A	A	A	A	A	A
17952	ASFV G ACD 00300	T	C	T	T	T	T	T	T	T
19791	Intergenic region	AGG	A	A	AG	AG	AG	AG	AG	AG
19998	ASFV G ACD 00350	GGGGG	GG	GGG	G	G	GG	GG	GG	G
21786	IR	CA	C	CA	CA	CA	CA	CA	CA	CA
21796	IR	C	CGGG	CGG	CG	CGG	CGG	CGG	CG	CG
27423	IR	TT	TT	TT	TT	TT	T	T	TT	TT
27619	MGF-360–11L	T	T	T	T	C	T	T	T	T
37442	MGF 505–4R	G	G	G	A	G	A	A	G	G
44576	MGF-505–9R	A	G	G	G	G	G	G	G	G
50112	IR	G	G	G	G	G	G	A	G	G
64736	K205R	G	G	G	A	A	A	A	G	G
70011	EP1242L	G	A	A	G	G	G	G	G	G
73262	IR	CT	CT	CT	CT	CT	C	C	CT	CT
74242	EP153R	A	A	A	A	A	A	A	A	AAAACAA
74327	IR	C	CTA	C	C	C	C	C	C	C
78536	M1249L	C	C	C	C	C	C	T	C	C
83868	C717R	T	T	T	T	T	T	T	T	G
97867	B438L	G	G	G	G	G	A	G	G	G
100436	B475L	TACTTATAAC	T	TACTTATAAC	TACTTATAAC	TACTTATAAC	TACTTATAAC	TACTTATAAC	TACTTATAAC	TACTTATAAC
103315	IR	G	GG	GG	GG	GG	G	G	GG	GG
109854	B263R	T	T	T	C	T	T	T	T	T
116162	G1211R	C	C	C	C	C	C	T	C	C
129418	O174L	G	G	G	G	G	G	G	A	G
129552	IR	G	G	G	G	G	G	G	A	G

Position per GA/2007	Region	GA/2007 (FR682468.2)	Ind/MZ/WB/324	Ind/MZ/314	Ind/AR/SD-61/2020 (OL692744)	Ind/AS/SD2/2020 (OL692743)	Ind/ABTCVSCK ASF001 (OM481275)	Ind/ABTCVSCK (OM481276)	China/2018/AnhuiXCGQ (MK128995)	Wuhan 2019–1 (MN393476)
129547	IR	G	G	G	G	G	G	G	A	G
130001	IR	CATATAAACGCATGC	CATATAAACGCATGC	C	CATATAAACGCATGC	CATATAAACGCATGC	CATATAAACGCATGC	CATATAAACGCATGC	CATATAAACGCATGC	CATATAAACGCATGC
134514	NP419L	T	C	C	C	C	C	C	C	C
167129	E199L	T	A	A	T	T	T	T	T	T
168750	IR	T	TA	TA	T	T	T	T	T	T
170731	I267L	C	C	C	CT	C	C	C	C	C
170862	I267L	T	A	A	A	A	A	A	A	A
173381	IR	G	GTATATAGGAA	GTATATAGGAA	GTATATAGGAA	GTATATAGGAA	GTATATAGGAA	GTATATAGGAA	GTATATAGGAA	GTATATAGGAA
174630	IR	C	T	T	C	C	C	C	C	C
176021	I196L	AT	A	AT	AT	AT	AT	AT	AT	AT
188048	MGF 360–21R	A	A	A	A	A	A	A	A	C
188949	MGF 360–21R	A	Deletion of 50 nt	A	A	A	A	A	A	A
189843	ASFV G ACD 01990	C	A	A	C	C	C	C	C	C
190372	3′ITR	C	CA	CA	CA	CA	CA	CA	C	CA

*IR, intergenic region.

Appendix Table 2. In-house designed primers used for gap-filling by Sanger sequencing

Primer name	5′→3′	No. of bases
ASF 176023F	ATAAGCCCATTAACACTAAGA	21
ASF 176361R	ACCTTTTTTATTCAACCTAAT	21
ASF 74392F	TCACGAATCTAATTATTGGGT	21
ASF 74865R	GTATCAAATACATCATTATGA	21
ASF 130031F	TGGCACTTGATGGTTCAAGTG	21
ASF 130379R	GACCATGTACTCTGACATCAT	21
ASF 175929F	ATTATATTCGAATGTTTGTCCA	22
ASF 176446R	AGTGATATTCCACTCTGATAC	21
ASF 189100F	GAAGACAGCATGGAACTAGCA	21
ASF 189371R	ATAATTATGTTACGTCATATA	21

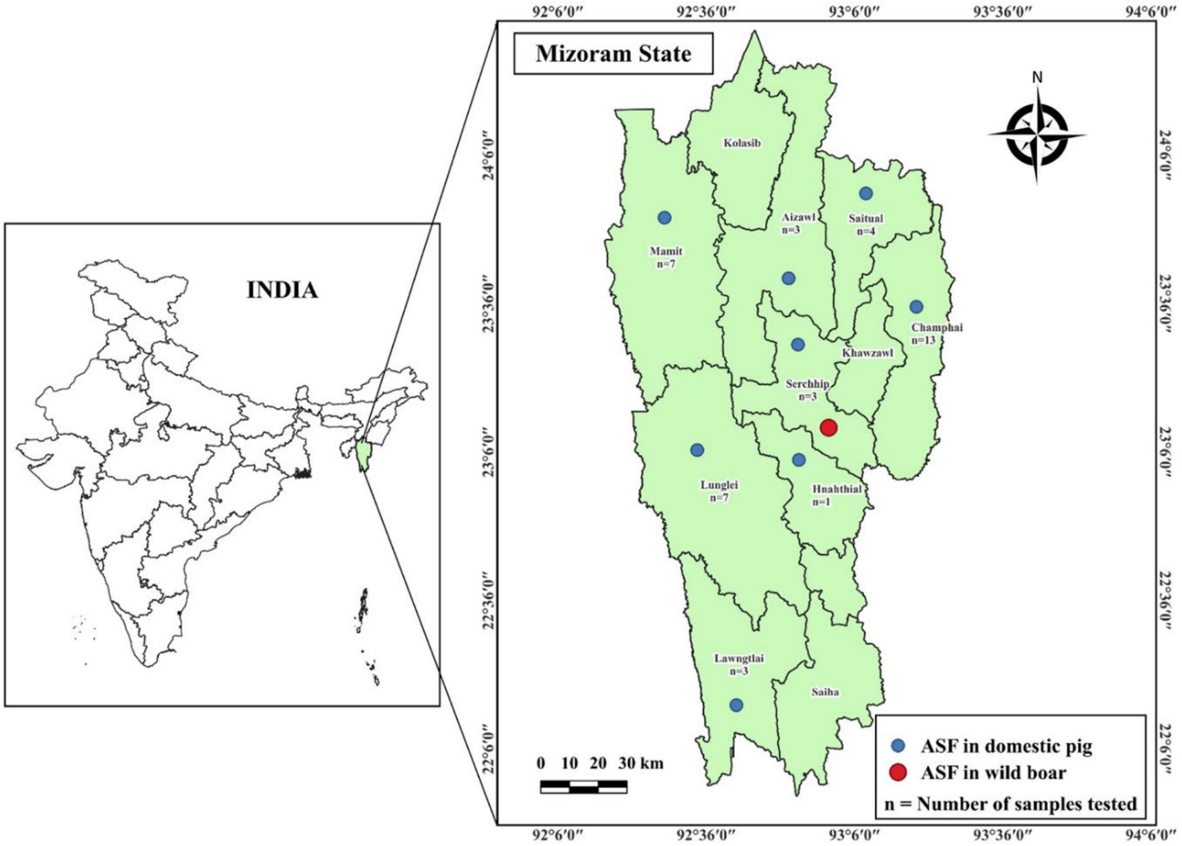

Appendix Figure 1. Map of Mizoram state highlighting the districts sampled during the African swine fever outbreaks, along with the number of samples tested for ASFV detection in this study.

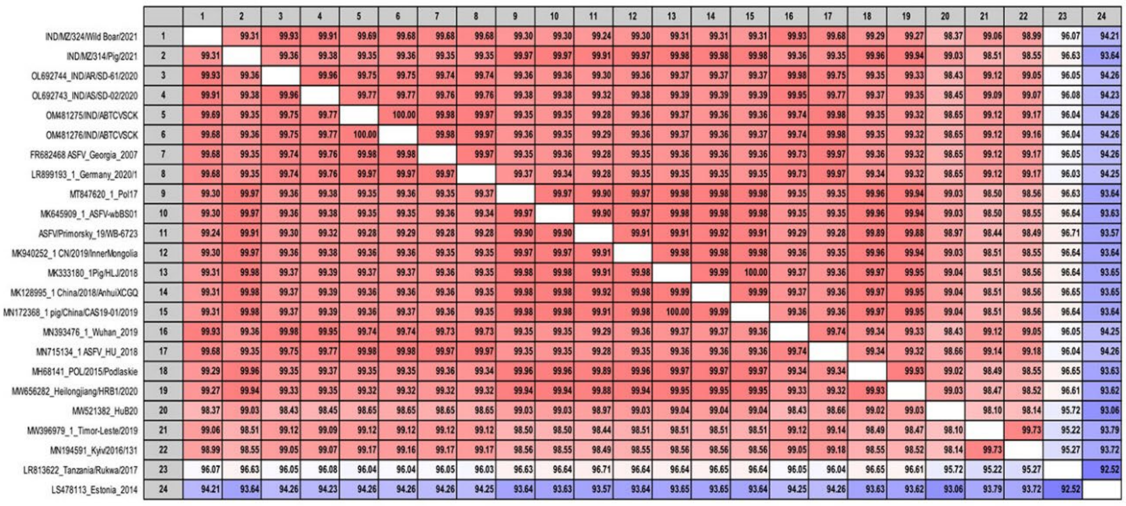

		1	2	3	4	5	6	7	8	9	10	11	12	13	14	15	16	17	18	19	20	21	22	23	24
IND/MZ/324/Wild Boar/2021	1		99.31	99.93	99.91	99.69	99.68	99.68	99.68	99.30	99.30	99.24	99.30	99.31	99.31	99.31	99.93	99.68	99.29	99.27	98.37	99.06	98.99	96.07	94.21
IND/MZ/314/Pig/2021	2	99.31		99.36	99.38	99.35	99.36	99.35	99.35	99.97	99.97	99.91	99.97	99.98	99.98	99.98	99.36	99.35	99.96	99.94	99.03	98.51	98.55	96.63	93.64
OL692744_IND/AR/SD-61/2020	3	99.93	99.36		99.96	99.75	99.75	99.74	99.74	99.36	99.36	99.30	99.36	99.37	99.37	99.37	99.98	99.75	99.35	99.33	98.43	99.12	99.05	96.05	94.26
OL692743_IND/AS/SD-02/2020	4	99.91	99.38	99.96		99.77	99.77	99.76	99.76	99.38	99.38	99.32	99.38	99.39	99.39	99.39	99.95	99.77	99.37	99.35	98.45	99.09	99.07	96.08	94.23
OM481275/IND/ABTCVSCK	5	99.69	99.35	99.75	99.77		100.00	99.98	99.97	99.35	99.35	99.28	99.36	99.37	99.36	99.36	99.74	99.98	99.35	99.32	98.65	99.12	99.17	96.04	94.26
OM481276/IND/ABTCVSCK	6	99.68	99.36	99.75	99.77	100.00		99.98	99.97	99.36	99.35	99.29	99.36	99.37	99.36	99.37	99.74	99.98	99.35	99.32	98.65	99.12	99.16	96.04	94.26
FR682468 ASFV_Georgia_2007	7	99.68	99.35	99.74	99.76	99.98	99.98		99.97	99.35	99.36	99.28	99.35	99.36	99.36	99.36	99.73	99.97	99.36	99.32	98.65	99.12	99.17	96.05	94.26
LR899193_1_Germany_2020/1	8	99.68	99.35	99.74	99.76	99.97	99.97	99.97		99.37	99.34	99.28	99.35	99.35	99.35	99.35	99.73	99.97	99.34	99.32	98.65	99.12	99.17	96.03	94.25
MT847620_1_Pol17	9	99.30	99.97	99.36	99.38	99.35	99.36	99.35	99.37		99.97	99.90	99.97	99.98	99.98	99.98	99.35	99.35	99.96	99.94	99.03	98.50	98.56	96.63	93.64
MK645909_1_ASFV-wbBS01	10	99.30	99.97	99.36	99.38	99.35	99.35	99.36	99.34	99.97		99.90	99.97	99.98	99.98	99.98	99.35	99.35	99.96	99.94	99.03	98.50	98.55	96.64	93.63
ASFV/Primorsky_19/WB-6723	11	99.24	99.91	99.30	99.32	99.28	99.29	99.28	99.28	99.90	99.90		99.91	99.91	99.92	99.91	99.29	99.28	99.89	99.88	98.97	98.44	98.49	96.71	93.57
MK940252_1 CN/2019/InnerMongolia	12	99.30	99.97	99.36	99.38	99.36	99.36	99.35	99.35	99.97	99.97	99.91		99.98	99.98	99.98	99.36	99.35	99.96	99.94	99.03	98.51	98.55	96.64	93.64
MK333180_1Pig/HLJ/2018	13	99.31	99.98	99.37	99.39	99.37	99.37	99.36	99.35	99.98	99.98	99.91	99.98		99.99	100.00	99.37	99.36	99.97	99.95	99.04	98.51	98.56	96.64	93.65
MK128995_1 China/2018/AnhuiXCGQ	14	99.31	99.98	99.37	99.39	99.36	99.36	99.36	99.35	99.98	99.98	99.92	99.98	99.99		99.99	99.37	99.36	99.97	99.95	99.04	98.51	98.56	96.65	93.65
MN172368_1 pig/China/CAS19-01/2019	15	99.31	99.98	99.37	99.39	99.36	99.37	99.36	99.35	99.98	99.98	99.91	99.98	100.00	99.99		99.36	99.36	99.97	99.95	99.04	98.51	98.56	96.64	93.64
MN393476_1_Wuhan_2019	16	99.93	99.36	99.98	99.95	99.74	99.74	99.73	99.73	99.35	99.35	99.29	99.36	99.37	99.37	99.36		99.74	99.34	99.33	98.43	99.12	99.05	96.05	94.25
MN715134_1 ASFV_HU_2018	17	99.68	99.35	99.75	99.77	99.98	99.98	99.97	99.97	99.35	99.35	99.28	99.35	99.36	99.36	99.36	99.74		99.34	99.32	98.66	99.14	99.18	96.04	94.26
MH68141_POL/2015/Podlaskie	18	99.29	99.96	99.35	99.37	99.35	99.35	99.36	99.34	99.96	99.96	99.89	99.96	99.97	99.97	99.97	99.34	99.34		99.93	99.02	98.49	98.55	96.65	93.63
MW656282_Heilongjiang/HRB1/2020	19	99.27	99.94	99.33	99.35	99.32	99.32	99.32	99.32	99.94	99.94	99.88	99.94	99.95	99.95	99.95	99.33	99.32	99.93		99.03	98.47	98.52	96.61	93.62
MW521382_HuB20	20	98.37	99.03	98.43	98.45	98.65	98.65	98.65	98.65	99.03	99.03	98.97	99.03	99.04	99.04	99.04	98.43	98.66	99.02	99.03		98.10	98.14	95.72	93.06
MW396979_1_Timor-Leste/2019	21	99.06	98.51	99.12	99.09	99.12	99.12	99.12	99.12	98.50	98.50	98.44	98.51	98.51	98.51	98.51	99.12	99.14	98.49	98.47	98.10		99.73	95.22	93.79
MN194591_Kyiv/2016/131	22	98.99	98.55	99.05	99.07	99.17	99.16	99.17	99.17	98.56	98.55	98.49	98.55	98.56	98.56	98.56	99.05	99.18	98.55	98.52	98.14	99.73		95.27	93.72
LR813622_Tanzania/Rukwa/2017	23	96.07	96.63	96.05	96.08	96.04	96.04	96.05	96.03	96.63	96.64	96.71	96.64	96.64	96.65	96.64	96.05	96.04	96.65	96.61	95.72	95.22	95.27		92.52
LS478113_Estonia_2014	24	94.21	93.64	94.26	94.23	94.26	94.26	94.26	94.25	93.64	93.63	93.57	93.64	93.65	93.65	93.64	94.25	94.26	93.63	93.62	93.06	93.79	93.72	92.52	

Appendix Figure 2. Pairwise comparison of nucleotide identity of ASFV isolated from wild boar with other Indian isolates, along with important genotype II isolates from other regions worldwide.

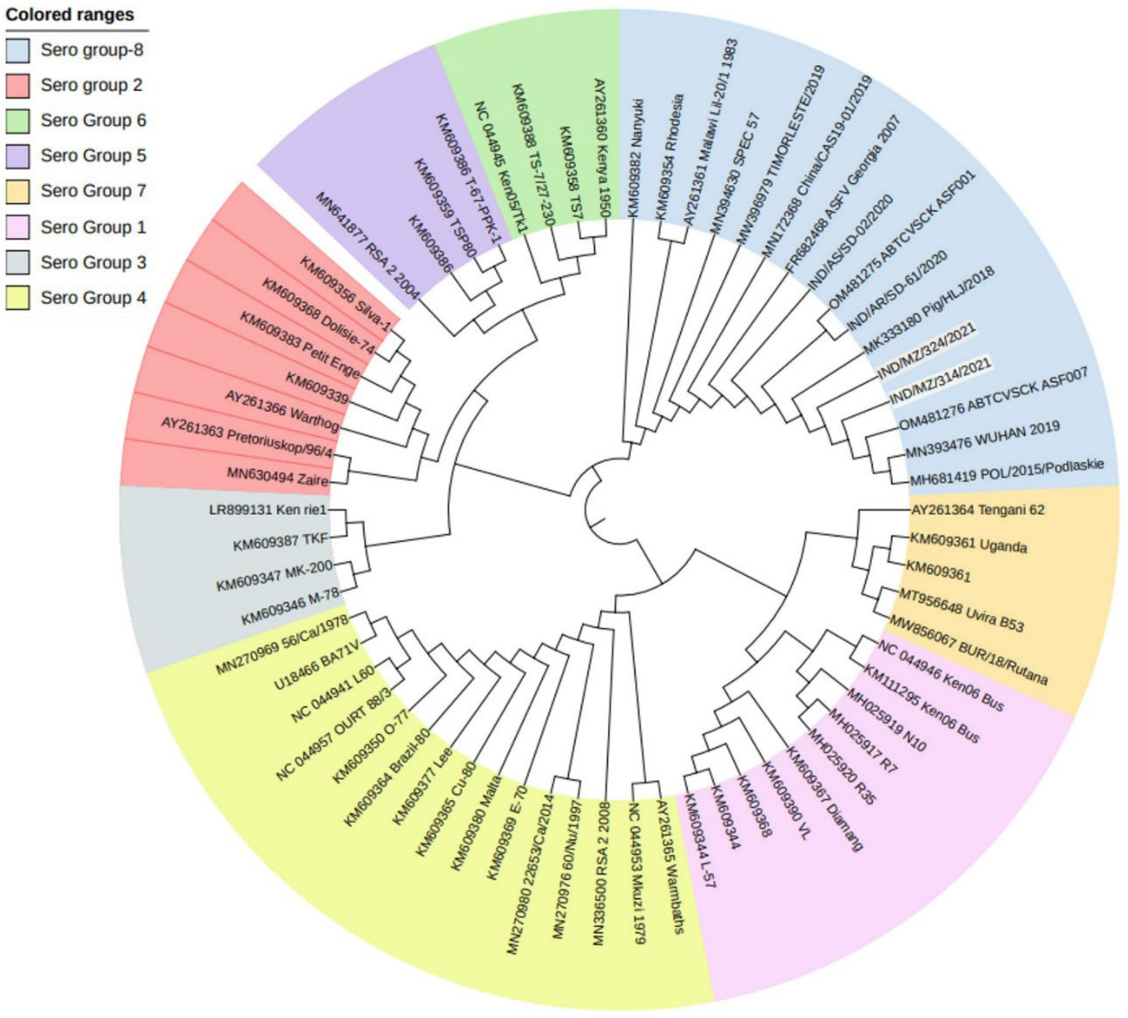

Appendix Figure 3. Maximum likelihood (ML) phylogenetic tree of ASFV sequences based on the EP402R gene. Branch lengths indicate the expected substitutions per site, illustrating serogrouping and evolutionary relationships among ASFV isolates.

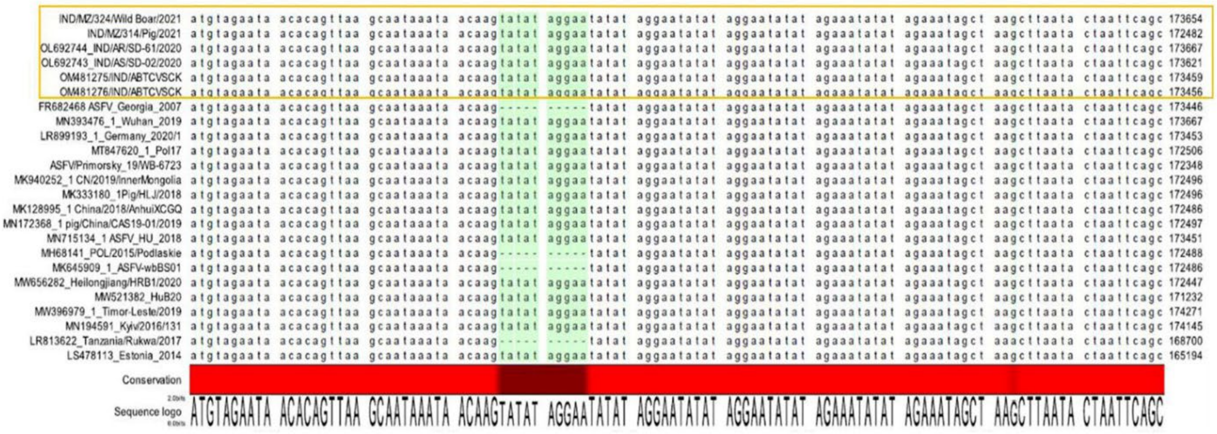

Appendix Figure 4. Insertion of one extra tandem repeat sequence (TRS) in the extragenic region between I73R and I329L compared with Georgia/2007 ASFV.

Gene name	5' ITR ASFV ACD 1990	ASFV ACD 300	MGF 360-11L	MGF 505-4R	MGF 505 9R	K205R	EP 1242L	MI249L	C717R	B438L	B263R	G1211R	O174L	O174L	O174L	NP419L	E199L	I267L	MGF 360-21R	3' ITR ASFV G ACD 01990
Nucleotide position in the gene	42	94	881	673	967	563	1217	1893	797	419	503	2005	199	224	328	1241	312	583	71	42
Amino acid Position (Variant <Indian isolates)	*	32 (K<E)	294 (E<G)	225 (K<E)	323 (K<E)	188 (R<K)	406 (P<L)	*	266 (G<V)	*	168 (V<A)	669 (F<L)	67 (S<P)	75 (S<F)	110 (S<P)	414 (N<S)	104 (Q<H)	195 (I<F)	24 (P<H)	*
IND/MZ/324/2021	T	G	A	G	G	G	A	C	T	G	T	C	C	C	C	G	T	T	A	T
IND/MZ/314/2021	-	A	A	G	G	G	A	C	T	G	T	C	C	C	C	G	T	T	A	T
OL692743/IND/AS/SD-02/2020	G	A	G	G	G	A	G	C	T	G	T	C	C	C	C	G	A	T	A	G
OL692744/IND/AR/SD-61/2020	G	A	A	A	G	A	G	C	T	G	C	C	C	C	C	G	A	T	A	G
OM481275/IND/ABTCVSCK	G	A	A	A	G	A	G	C	T	G	T	T	C	C	C	G	A	T	A	G
OM481276/IND/ABTCVSCK	G	A	A	A	G	A	G	T	T	A	T	C	C	C	C	G	A	T	A	G
Georgia/2007	G	A	A	G	A	G	G	C	T	G	T	C	C	C	C	A	A	T	A	G
Anhui XCGQ/2018	-	A	A	G	G	G	G	C	T	G	T	C	T	T	T	G	A	T	A	G
Wuhan/2019-1	G	A	A	G	G	G	G	C	G	G	T	C	C	C	C	G	A	A	C	G

*- indicates synonymous mutation, Red indicates unique SNPs present only in the ASFV isolate from the wild boar, while yellow highlights SNPs shared by both Mizoram isolates in comparison to other Indian isolates included in this study.

Appendix Figure 5. Single-nucleotide polymorphism (SNP) analysis of ASFV isolates from wild boar and domestic pig in Mizoram compared with previously reported Indian isolates and other significant ASFV strains globally.
